# Impact of surgeon specialty on clinical outcomes following esophagectomy for cancer

**DOI:** 10.1007/s00464-023-10391-5

**Published:** 2023-09-07

**Authors:** Arjun Verma, Joseph Hadaya, Elsa Kronen, Sara Sakowitz, Nikhil Chervu, Syed Shahyan Bakhtiyar, Peyman Benharash

**Affiliations:** 1 Cardiovascular Outcomes Research Laboratories (CORELAB), University of California, Los Angeles, Los Angeles, CA USA; 2 Department of Surgery, University of California, Los Angeles, Los Angeles, CA USA; 3 Department of Surgery, University of Colorado Anschutz Medical Center, Aurora, CO USA; 4 Division of Cardiac Surgery, Department of Surgery, University of California, Los Angeles, Los Angeles, CA USA

**Keywords:** Esophagectomy, Esophageal cancer, Surgeon specialty, ACS NSQIP, Clinical outcomes

## Abstract

**Background:**

The impact of surgeon and hospital operative volume on esophagectomy outcomes is well-described; however, studies examining the influence of surgeon specialty remain limited. Therefore, we evaluated the impact of surgeon specialty on short-term outcomes following esophagectomy for cancer.

**Methods:**

The 2016–2019 American College of Surgeons National Surgical Quality Improvement Project (ACS NSQIP) was queried to identify all patients undergoing esophagectomy for esophageal cancer. Surgeon specialty was categorized as general (GS) or thoracic (TS). Entropy balancing was used to generate sample weights that adjust for baseline differences between GS and TS patients. Weights were subsequently applied to multivariable linear and logistic regressions, which were used to evaluate the independent association of surgeon specialty with 30-day mortality, complications, and postoperative length of stay.

**Results:**

Of 2657 esophagectomies included for analysis, 54.1% were performed by TS. Both groups had similar distributions of age, sex, and body mass index. TS patients more frequently underwent transthoracic esophagectomy, while GS patients more commonly received minimally invasive surgery. After adjustment, surgeon specialty was not associated with altered odds of 30-day mortality (adjusted odds ratio [AOR] 1.10 *p* = 0.73) or anastomotic leak (AOR 0.87, *p* = 0.33). However, TS patients exhibited a 40-min reduction in operative duration and faced greater odds of perioperative transfusion, relative to GS.

**Conclusion:**

Among ACS NSQIP participating centers, surgeon specialty influenced operative duration and blood product utilization, but not mortality and anastomotic leak. Our results support the relative safety of esophagectomy performed by select GS and TS.

Despite incremental refinements in patient selection, surgical technique, and perioperative management, esophagectomy remains a complex operation that is associated with major morbidity in approximately 17% of patients [1]. Patient characteristics linked to mortality and complications have been extensively delineated; however, recent investigations have focused on potentially modifiable factors related to surgical expertise [2–4]. Importantly, a large body of literature has demonstrated case volume to be an acceptable marker of quality, with lower rates of mortality and complications at centers performing a high volume of complex procedures [5, 6].

A less commonly explored facet of expertise is specialized surgical training [7–9]. While it is generally thought that additional training results in superior outcomes, pragmatic issues with access, insurance coverage, and surgeon availability often preclude the performance of many complex operations exclusively by specialty trained surgeons. In fact, a study by Khoushhal and colleagues demonstrated that over 70% of esophageal resections are performed by general surgeons, with superior outcomes for patients treated by thoracic surgeons [10]. However, the authors examined a dated patient population that may no longer reflect the landscape of esophagectomies in the current era. Contemporary studies examining the pragmatic influence of specialized thoracic surgery training on esophagectomy outcomes are also limited by lack of adjustment for relevant covariates and use of non-robust statistical techniques [10–12].

Therefore, the present study analyzed a large cohort of patients undergoing esophagectomy for cancer and used robust statistical methods to evaluate the association of surgeon specialty with mortality, complications, and postoperative length of stay. We hypothesized similar outcomes between patients managed by thoracic and general surgeons, but significantly different distribution of operative approach.

## Methods

This was a retrospective cohort study of the 2016–2019 American College of Surgeons National Surgical Quality Improvement Program (ACS NSQIP) participant use files. The ACS NSQIP and the hospitals participating in the ACS NSQIP are the source of the data used herein; they have not verified and are not responsible for the statistical validity of the data analysis or the conclusions derived by the authors. Given the de-identified nature of the ACS NSQIP, this study was deemed exempt from full review by the Institutional Review Board at the University of California, Los Angeles.

All adults undergoing elective esophagectomy for cancer were identified using relevant *Current Procedural Terminology* codes [13]. Records with missing data for age, sex, race, elective/urgent/emergency status, and surgeon specialty were excluded (< 1%). Surgeon specialty was categorized into general (GS) and thoracic (TS). The ACS NSQIP defines surgeon specialty as the Division/Department of the primary surgeon [12]. It is important to note that this definition is broad and does not provide any information about training history, including type of residency (general surgery/integrated cardiothoracic) or fellowship (minimally invasive surgery, surgical oncology, hepato–pancreato–biliary). Given that only the primary surgeon’s specialty is recorded, jointly performed cases could not be separately analyzed.

Demographic and clinical variables were defined using ACS NSQIP-provided data elements and included age, female sex, body mass index, diabetes, hypertension, smoking status, chronic obstructive pulmonary disease (COPD), chronic steroid use, weight loss, pre-existing bleeding disorder, functional status, and American Society of Anesthesiologists (ASA) Physical Status Classification System [14]. Operation type was stratified into transhiatal and transthoracic, while surgical approach was categorized into open and minimally invasive (laparoscopic, thoracoscopic, and/or robotic assisted). Anastomotic leak, pneumonia, positive margins, and intra/postoperative blood transfusion as well as cardiac (myocardial infarction, cardiac arrest), thromboembolic (deep vein thrombosis and pulmonary embolism), and infectious (tissue/organ infection, pneumonia, urinary tract infection, sepsis, and septic shock) complications were ascertained. The primary endpoint was 30-day mortality, while operative duration, complications, and postoperative length of stay (LOS) were secondarily assessed.

Categorical variables are reported as percentages (%), and continuous variables are reported as medians with interquartile range (IQR). The Pearson’s chi-squared and Mann–Whitney *U* tests were used for unadjusted comparisons. We used entropy balancing as the first-line statistical approach to mitigate the influence of significant intergroup differences. Briefly, this reweighing method provides a balanced distribution of covariates and has been shown to be superior to propensity matching [15, 16]. Following application of entropy balancing-derived weights, multivariable logistic and linear regressions were developed to evaluate the association between surgeon specialty and outcomes of interest. As a sensitivity analysis, multivariable logistic and linear regression models were developed without entropy balancing to reassess the association of surgeon specialty with outcomes of interest. Variable selection was performed using elastic net regularization, which combines the Least Absolute Shrinkage and Selection Operator and Ridge regression penalties to reduce bias and increase generalizability [17]. Regression outputs are reported as adjusted odds ratios (AOR) or beta coefficients (*β*) with 95% confidence intervals (95% CI). Statistical significance was set at an α of 0.05. All statistical analysis was performed using Stata 16.1 (StataCorp, College Station, TX).

## Results

Of 2657 esophageal resections included for analysis, 1437 (54.1%) were performed by TS. The proportion of patients managed by TS remained steady over the 4-year study period, at 54.2% in 2016 and 53.9% in 2019 (nptrend = 0.79). Patients treated by GS and TS had similar distributions of age (GS: 44.8 vs TS: 44.5 years, *p* = 0.43), female sex (17.6 vs 17.4%, *p* = 0.88), and BMI (27.7 vs 27.6 kg/m^2^, *p* = 0.39). Moreover, both groups had comparable preoperative ASA Physical Status Classification, as shown in Table 1. The incidence of all studied comorbidities, including diabetes, hypertension, and COPD, were equivalent between cohorts, with the notable exception of chronic steroid use, which was higher among TS patients (3.1 vs 1.7%, *p* = 0.026). Neoadjuvant chemotherapy and radiation rates were comparable between groups, as were clinical T, N, and M stage (Table 1). TS patients were more commonly diagnosed with squamous cell carcinoma, and less frequently adenocarcinoma. Of note, TS patients more frequently underwent transthoracic esophagectomy (89.8 vs 64.7%, *p* < 0.001), compared to GS. However, GS patients were more commonly managed using minimally invasive techniques (67.0 vs 47.7%, *p* < 0.001).

**Table 1:** Comparison of baseline characteristics between patients of thoracic (TS) and general (GS) surgeons

Parameter	GS *n* = 1220	TS *n* = 1437	*p* value
Age (years, mean ± SD)	44.8 ± 9.3	44.5 ± 9.1	0.43
Female sex (%)	17.6	17.4	0.88
Body mass index (kg/m^2^, mean ± SD)	27.7 ± 5.6	27.6 ± 5.9	0.39
Minimally invasive operation (%)	67.0	47.7	< 0.001
Operation type (%)			< 0.001
Transthoracic	64.7	89.8	
Transhiatal	35.3	10.2	
ASA class (%)			0.044
I	0.33	0.14	
II	17.8	14.1	
III	74.6	78.6	
IV	7.3	7.2	
Neoadjuvant therapy (%)			
Chemotherapy	68.9	69.0	0.96
Radiation	59.0	56.7	0.23
Clinical T stage (%)			0.055
T0	0.9	0.5	
T1	11.5	9.3	
T2	15.4	15.0	
T3	49.9	49.3	
T4	0.9	2.0	
Unknown	21.4	24.0	
Clinical N stage (%)			0.21
N0	35.4	30.4	
N1	28.0	29.0	
N2	9.1	8.5	
N3	1.0	1.3	
Unknown	26.6	30.8	
Clinical M stage (%)			0.71
M0/Mx	71.1	60.8	
M1	1.0	1.0	
Unknown	28.0	38.2	
Pathology (%)			< 0.001
Adenocarcinoma	86.5	82.7	
Dysplasia	1.7	4.0	
Squamous cell carcinoma	8.8	10.8	
Other/unknown	3.0	2.6	
Comorbidities (%)			
Diabetes			0.059
None	82.6	79.3	
No Insulin	10.9	13.9	
Insulin	6.8	6.9	
Hypertension	48.8	48.4	0.86
History of smoking	23.0	27.6	0.008
Chronic obstructive pulmonary disease	7.8	8.0	0.84
Chronic steroid use	1.7	3.1	0.026
Preoperative weight loss	21.6	21.7	0.96
Metastatic disease	3.4	4.7	0.11
Bleeding disorder	4.0	3.8	0.73

*SD* standard deviation, *ASA* American Society of Anesthesiologists

Upon unadjusted analysis, GS and TS patients faced similar rates of 30-day mortality (GS: 2.6 vs TS: 3.7%, *p* = 0.12). Moreover, the occurrence of postoperative pneumonia and anastomotic leak were comparable between groups, as were rates of cardiac, thromboembolic, and infectious complications (Table 2). Patients within the GS and TS cohorts had equivalent rates of residual tumor. TS patients more frequently required intra/postoperative blood transfusions (13.7 vs 8.9%, *p* < 0.001) but had shorter mean operative times compared to GS (345 vs 375 min, *p* < 0.001). Postoperative length of stay was equivalent between cohorts.

**Table 2:** Unadjusted and risk-adjusted comparison of outcomes between thoracic (TS) and general (GS) surgeons

Outcome	GS *n* = 1220	TS *n* = 1437	*p* value	AOR/*ß*	95% CI	*p* value
30-Day mortality (%, AOR)	2.6	3.7	0.12	1.10	0.64–1.89	0.73
Pneumonia (%, AOR)	12.9	13.6	0.60	0.83	0.64–1.09	0.19
Anastomotic leak (%, AOR)	13.3	12.5	0.53	0.87	0.66–1.15	0.33
Cardiac complication (%, AOR)	2.8	2.5	0.83	1.01	0.57–1.82	0.94
Thromboembolic complication (%, AOR)	3.9	4.1	0.82	0.93	0.59–1.48	0.77
Infectious complication (%, AOR)	26.7	27.0	0.87	0.84	0.69–1.03	0.099
Positive margins (%, AOR)	7.9	8.1	0.85	0.91	0.66–1.26	0.58
Intra/postoperative transfusion (%, AOR)	8.9	13.7	< 0.001	1.44	1.05–1.96	< 0.001
Operative duration (minutes, mean ± SD, ß)	375 ± 136	345 ± 124	< 0.001	− 40	− 51, − 28	< 0.001
Postoperative LOS (days, mean ± SD, ß)	11.3 ± 8.5	11.6 ± 8.7	0.32	− 0.5	− 1.4, 0.4	0.24

Risk-adjustment was performed using entropy balancing

*LOS* length of stay, *CI* confidence interval, *SD* standard deviation, *AOR* adjusted odds ratio, ß ß-coefficient

**Fig. 1:**
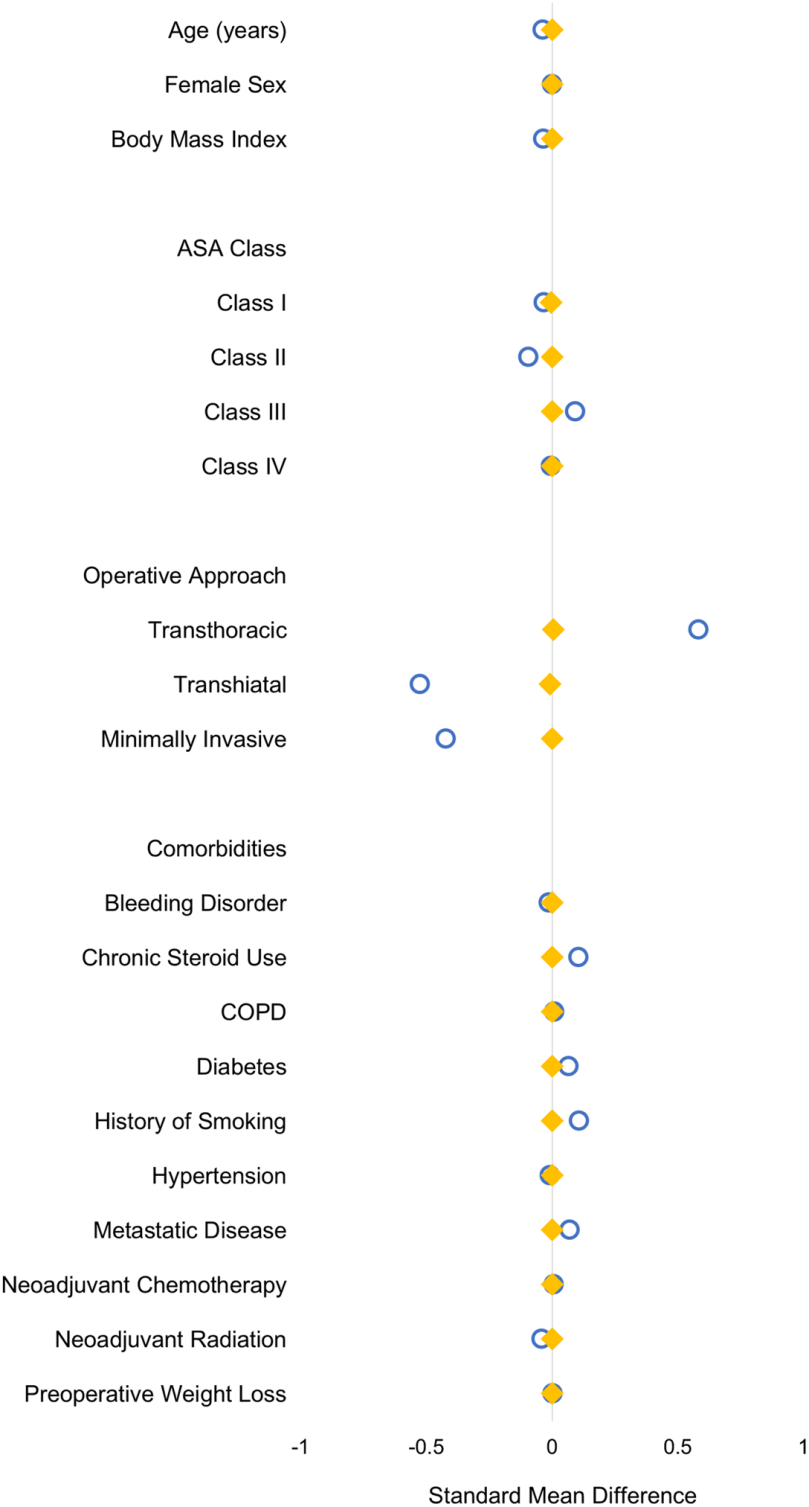
Distribution of patient and operative characteristics before and after entropy balancing. Blue represents pre-balancing and yellow represents post-balancing (Color figure online)

Application of entropy balancing resulted in comparable distribution of covariates between GS and TS patients (Fig. 1). After adjustment, surgeon specialty was not associated with altered odds of 30-day mortality (AOR 1.10, 95% CI 0.64–1.89) or anastomotic leak (AOR 0.87, 95% CI 0.66–1.15) as well as cardiac, thromboembolic and infectious complications (Table 2). Notably, TS patients had similar odds of developing pneumonia (AOR 0.83, 95% CI 0.64–1.09) and having positive margins (AOR 0.91, 95% CI 0.66–1.26) but faced a 44% increment in relative odds of intra/postoperative transfusion (Table 2). Moreover, thoracic surgical specialty was associated with a 40-min reduction in operative duration (95% CI − 51, − 28), with GS as reference. Surgeon specialty was not associated with postoperative LOS.

**Fig. 2:**
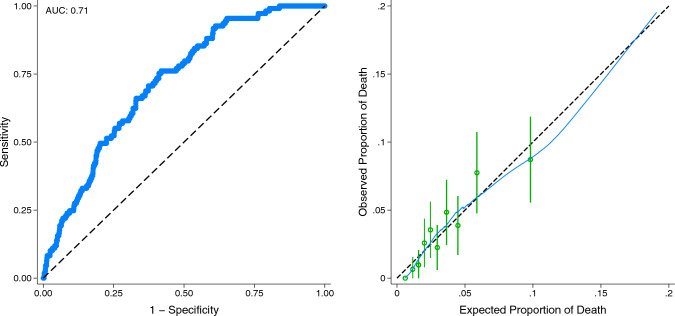
Area under the receiver operating characteristic curve and reliability plot of the multivariable model to predict 30-day mortality

Following multivariable modeling without application of entropy balancing weights, surgeon specialty remained unassociated with 30-day mortality (AOR 1.21, 95% CI 0.74–1.98). Additional factors associated with mortality included increasing age (AOR 1.06/year, 95% CI 1.03–1.09) and ASA Class IV (AOR 3.86, 95% CI 1.29–11.47, ref Class II). The area under the receiver operating characteristic curve and reliability plot for this model are shown in Fig. 2. Furthermore, relative to GS, TS continued to display no association with the likelihood of anastomotic leak, pneumonia, and positive margins, as well as cardiac, thromboembolic, and infectious complications. However, patients managed by TS had greater odds of intra/postoperative transfusion (AOR 1.44, 95% CI 1.09–1.90) and faced shorter operation times, with an average reduction of 41 min (95% CI − 50, − 32).

## Discussion

Esophagectomy for primary esophageal malignancy remains a complex operation requiring expertise in both thoracic and foregut surgery. In the present study, we assessed whether short-term clinical outcomes following esophagectomy were associated with surgeon specialty. Following adjustment for relevant confounders, we found no significant difference in 30-day mortality and most complications between general and thoracic surgeons. Of note, thoracic surgeons more commonly employed transthoracic approaches and had shorter operative times, while general surgeons more frequently used minimally invasive techniques and less often required intra/postoperative blood transfusion. Several of our findings warrant further discussion.

In the United States, esophagectomy is performed by thoracic and general surgeons, with practice patterns varying across regions, healthcare networks, and institutions. Thoracic surgeons most commonly receive dedicated training in the management of esophageal cancer through cardiothoracic surgery fellowships, while general surgeons can obtain greater expertise in esophageal malignancies through surgical oncology or minimally invasive surgery programs. Congruent with prior reports, we found that general and thoracic surgeons had equivalent rates of mortality and most complications [11, 12, 18]. This observation is likely related to appropriate case selection by surgeons in both specialties. Indeed, our sample represents cases performed at ACS NSQIP-participating hospitals, which may have general surgeons that are more experienced compared to those at non-NSQIP participating facilities [19]. Similarly, given that the majority of deaths following major operations occur in the postoperative phase, it is plausible that institution-specific care pathways allow for improved care and lower failure-to-rescue rates, thereby mitigating the influence of surgeon specialty on short-term outcomes [6, 11, 20–22]. Importantly, we could not surmise whether the general surgeons in our study had specialized training (e.g., surgical oncology or minimally invasive surgery). In addition, we were unable to evaluate the influence of surgeon specialty on overall and tumor-free survival. As such, further study with granular, longitudinal data is required to further verify the safety of select general surgeons performing esophagectomy.

As expected, thoracic surgeons more commonly performed transthoracic esophagectomies, relative to general surgeons. The increased utilization of Ivor-Lewis and McKeown esophagectomy by thoracic surgeons likely reflects greater familiarity and training in chest surgery. Of note, most contemporary studies have reported comparable outcomes between transhiatal and transthoracic esophagectomy, leaving the choice of surgical approach highly dependent on surgeon, institution, patient, and tumor characteristics [23–25]. Efforts to identify the most appropriate surgical and medical care for each patient, such as multidisciplinary tumor boards, should be pursued at hospitals which have both general and thoracic surgeons performing esophagectomy.

Consistent with a prior report by Hsu et al., we found that operative duration was shorter among TS patients [12]. This observation is most attributable to inherent differences in practice settings of thoracic and general surgeons, wherein thoracic surgeons may have relatively greater esophagectomy volume than their general surgery counterparts. This hypothesis could not be tested within the ACS NSQIP due to the lack of surgeon identifiers. Similarly, altered transfusion rates between cohorts may reflect variations in blood product utilization practices acquired through different training settings or the presence of department/division specific transfusion protocols. In an analysis of over 7000 patients undergoing esophagectomy, Towe et al. found significant variation in transfusion practices across centers in the United States and attributed their findings to the presence of standardized protocols, surgeon-specific training histories, and center-level differences in operative volume [26]. Several studies have emphasized the relevance of surgeon and hospital volume on patient outcomes [5, 6, 11, 20–22]. For instance, Munasinghe et al. highlighted the clinical and financial benefits of centralizing esophagectomy care in the United States [27]. However, centralization may have significant detrimental effects, including reduced access to care and longer intervals between diagnosis and surgery for socioeconomically disadvantaged patients [23, 28, 29]. While our results may not be generalizable to non-NSQIP-participating centers, they suggest the relative safety of esophageal resections performed by adequately trained surgeons in both specialties. By increasing the pool of surgeons performing this operation, there will undoubtedly be marked increases in access to care for underserved communities.

The present work has several important limitations inherent to its retrospective design. Of note, the ACS NSQIP is derived from patient data collected at select, high volume, teaching hospitals. Therefore, our results are not generalizable to all centers in the country, where surgeon-specific differences in training, operative experience, and outcomes may exist. Additionally, granular information about training history, including completion of an integrated cardiothoracic surgery, surgical oncology or minimally invasive surgery program, is not available. Certain clinical characteristics were not available for analysis, including location of the tumor, type of transthoracic esophagectomy (Ivor Lewis/McKeown), and route of alimentary tract reconstruction. Finally, the ACS NSQIP does not record long-term patient outcomes, and thus, we were unable to study the influence of surgeon specialty on oncologic outcomes and long-term survival.

In summary, the present study analyzed a large sample of patients undergoing esophagectomy for cancer and found that surgeon specialty was not associated with 30-day mortality or most complications. Our results support the relative safety of esophagectomy performed by select general and thoracic surgeons and demonstrate the relevance of surgeon-specific analysis to improve quality of care.
